# This sounds important: Electrophysiological responses reveal a dedicated learning mechanism to process salient consonant sounds in human newborns

**DOI:** 10.3758/s13423-026-02911-w

**Published:** 2026-06-15

**Authors:** Paolo Barbieri, Pietro Sarasso, Alice Rossi-Sebastiano, Jacopo Frascaroli, Karol Poles, Chiara Peila, Alessandra Coscia, Francesca Garbarini, Irene Ronga

**Affiliations:** 1 BIP Research Group, Department of Psychology, University of Turin, Via Verdi 10, 10124 Turin, Italy; 2 Manibus Lab, Department of Psychology, University of Turin, Turin, Italy; 3 City of Health and Science of Turin, Neonatal Unit of the University, Turin, Italy

**Keywords:** Newborns, Mismatch responses, Bayesian surprise, Consonance

## Abstract

Isolating relevant sounds in the auditory stream is a crucial feat accomplished by human infants and a pivotal ability for language acquisition. It is therefore reasonable to postulate the existence of early mechanisms that reorient attention toward salient acoustic stimuli. Previous studies suggest that infants tend to allocate more attention to consonant sounds because they resemble human vocalizations. However, systematic evidence on the neural processes underlying consonance tuning in newborns is still scarce. Here, we investigate newborns’ learning of auditory regularities by computing mismatch responses (MMRs) and the trial-by-trial correlation between the neural signal and Bayesian surprise (a theoretical measure of learning). We present 22 healthy newborns (40.4 ± 15.8 h) with a roving paradigm (a pseudorandom sequence of deviant and standard events), composed of either consonant or dissonant sounds, while we record their electroencephalogram. Our results show that 1) newborns exhibit a neural encoding of auditory regularities for all sound types (consonant and dissonant), as demonstrated by the presence of MMRs and significant correlation of the neural signal with Bayesian surprise; 2) consonant and dissonant sounds elicited MMRs of opposite polarities, with consonant auditory stimulation evoking negative responses, reminiscent of adult-like MMRs. Overall, our findings suggest that newborns display a dedicated perceptual learning mechanism for consonance. We speculate that this mechanism might represent an evolutionarily acquired process to detect and learn novel auditory stimuli with acoustic features resembling human vocalizations.

**Supplementary Information:**

The online version contains supplementary material available at 10.3758/s13423-026-02911-w.

## Introduction

The ability to discriminate and track salient, informative sounds while being immersed in a rich and noisy sensory environment underpins many human capacities, including verbal communication (Cheng et al., 2015; Friedrich et al., 2009). One should therefore expect to find neural traces of the development of such a crucial ability early on in the lifespan.

Although findings are still mixed (Plantinga & Trehub, 2014), preliminary behavioral evidence suggests that newborns and infants consider consonant sounds more salient and allocate greater attention to them than to dissonant sounds (Bowling et al., 2017; Di Stefano et al., 2017; Masataka, 2006; Schellenberg & Trehub, 1996; Trainor & Heinmiller, 1998; Trehub, 2003; Zentner & Kagan, 1996). Consonance and dissonance are acoustic phenomena typically defined by their perceptual qualities. While consonance is usually experienced as smooth, stable, and pleasant, dissonance is often perceived as rough, unstable, and unpleasant (Di Stefano et al., 2022). Newborns’ behavioral attunement toward consonance is often interpreted in light of the vocal similarity theory (Bowling et al., 2018). Human voices produce sounds that are harmonic in structure and thus more similar to consonant musical intervals. Therefore, preferences for consonance may be driven by an evolutionary mechanism directed at enhancing the processing of sounds similar to the human voice, as this would favor the acquisition of communication skills, and thereby signal more salient stimuli and confer adaptive significance (Bowling et al., 2017, 2018).

Consistent with this hypothesis, in a recent study on adults (Sarasso et al., 2022a, 2022b), preferred consonant sounds were found to elicit enhanced learning mechanisms, as demonstrated by the magnification of the mismatch negativity. Such mismatch responses (from now on, MMRs) represent a well-validated neurobiological marker of learning (Garrido et al., 2016; Näätänen et al., 2007), and despite being fully implicit measures, they have been systematically related to explicit learning enhancements (Batterink et al., 2015; Moser et al., 2021). Unlike adults, newborns present two distinct MMRs with opposite polarities (Friederici et al., 2002; Virtala et al., 2013; Werwach et al., 2022). Deviancy detection can elicit positive (Werwach et al., 2022), and (more rarely) negative MMRs (Cheour et al., 2002; Kostilainen et al., 2018; Virtala et al., 2013), sharing similar latencies (between 200 and 450 ms) and scalp distributions (frontocentral). Several factors can modulate MMRs’ polarity, such as interval physical features (i.e., pitch, intensity, duration, and deviance magnitude) and methodological parameters (interstimulus interval, sound duration, cognitive load, frequency-filtering; Cheng et al., 2015; Friedrich et al., 2009). Although findings remain controversial, recent evidence suggests that this phenomenon may be better understood through an integrative perspective (Govaart et al., 2023) combining two different explanations: the *maturation hypothesis* and the *multiple-processes* hypothesis. The former suggests that the positive MMRs reflect immature auditory processing, which progressively develops into the more adult-like negative MMR with age (Werwach et al., 2022). The latter proposes that, even at an early developmental stage, positive MMRs are associated with bottom-up, immature acoustic processing, whereas negative MMRs reflect more refined auditory discriminations, particularly tuned to linguistically relevant stimuli (Garcia-Sierra et al., 2016; Govaart et al., 2023; Werwach et al., 2022). Accordingly, the integrative perspective (Govaart et al., 2023) posits the coexistence of both mechanisms, with the negative polarity of the MMR signaling the progressive maturation along the developmental trajectory (i.e., time), as well as the selective maturation of the processing of specific acoustic stimuli (such as consonance) at early stages of development. In any case, negative MMRs are more systematically observed at later stages of development (Chen et al., 2022; Choisdealbha et al., 2022; He et al., 2007; Werwach et al., 2022) and in response to native language stimuli, even in the first year of life (Cheour et al., 2002; Garcia-Sierra et al., 2016; Kostilainen et al., 2018). However, whether the early polarity switch of the MMRs may be related to the processing of more relevant consonant sounds is still unclear. Here, we aim to test whether MMRs’ polarity switch observed in newborns actually represents an early tool for differentiating consonant and dissonant stimuli, with the aim of detecting the most informative stimuli in the highly variable sensory environment.

In the present study, we explore how newborns encode sensory regularities and update these encodings in light of surprising sensory inputs (a process hereafter referred to as *perceptual learning*; see Lieder et al., 2013) by computing the MMRs. Moreover, we assess whether a specific perceptual learning mechanism, directed to process consonant sounds, is already present in the first hours of life. With these aims in mind, we perform a trial-by-trial correlation between the brain signal elicited by each auditory event and the output obtained through the mathematical computation of Bayesian surprise values. Bayesian surprise is a mathematical measure of how much new information is conveyed by each stimulus in a sequence (Faraji et al., 2018; Ostwald et al., 2012). The correlation of the neural signal with this measure describes the degree to which the representations of the sensory environment are updated in response to deviant stimuli (Mousavi et al., 2020). In other words, this correlation can be considered an index of how effectively we are learning from the environment. Interestingly, in adults, the amplitude of mismatch negativity correlates with the trial-by-trial fluctuations of Bayesian surprise, thus confirming that MMRs reflect perceptual learning dynamics (Sarasso et al., 2021, 2022a, 2022b). However, it is still unknown whether this correspondence between MMRs and trial-by-trial fluctuations of Bayesian surprise is already present at birth. More specifically, the present study represents the first electrophysiological investigation combining the use of a roving paradigm with the trial-by-trial computation of the correlation with Bayesian surprise in newborns, thereby providing an exploratory examination of implicit learning dynamics at this early stage of life.

In sum, in the present study we aim to 1) investigate newborns’ perceptual learning of auditory regularities in a roving paradigm, and test whether the brain signal elicited by each auditory event in the sequence correlates with Bayesian surprise values, and 2) verify the presence of a dedicated perceptual learning mechanism to process novel consonant versus dissonant stimuli, as measured by differences in MMRs and correlation with Bayesian surprise. As for the results, 1) we expect to observe MMRs in response to both consonant and dissonant intervals and a significant correlation between the trial-by-trial fluctuation of the neural signal and Bayesian surprise values, indicating an efficient attunement to the informativeness of the auditory stream; 2) we predict that consonant sounds will elicit negative MMRs, while dissonant sounds will elicit positive MMRs, providing evidence for the presence in newborns of an early neural mechanism capable of discriminatinge consonant stimuli that are similar to the human voice spectrum.

## Materials and methods

### Participants

In the present experiment, we recorded the electroencephalography (EEG) of 22 newborns (*M*_age_ = 40.4 ± 15.8 h at the time of testing) to compute event-related potentials (ERPs) in response to an auditory roving paradigm (Ostwald et al., 2012). Parents provided written informed consent and the Ethical Committee of the Obstetric University Hospital approved the study (14/12/2017–14/12/2022). The original sample size (*N* = 25) was a priori determined so as to match the average number of participants involved in previous ERP studies on newborns (Cheour et al., 2002; Ronga et al., 2021) and in a very similar paradigm on adults (Sarasso et al., 2022a, 2022b). Three newborns were excluded due to crying or excessive movement during the experiment, which led to highly artifact-contaminated recordings (i.e., more than half of the trials) and/or incomplete data acquisition. Newborns included in the final dataset (*N* = 22) were awake during the presentation of the experimental conditions and relaxed in their parents’ (mother or father) arms. Furthermore, four newborns were excluded from the sample of the Bayesian surprise analysis since we were not able to collect enough trials during the experiment to perform the trial-by-trial analysis (due to deep sleep or crying).

### Data availability

Raw data used in the study and the tables containing the mean amplitude used in the analysis are fully available at this link (Mendeley, https://data.mendeley.com/preview/8vcx3ygxtw?a=fd2fe388-7524-404a-b37b-99b65cf0438c). All the data collected for the present research will be made publicly available upon acceptance for publication.

### Stimuli and experimental design

**Fig. 1:**
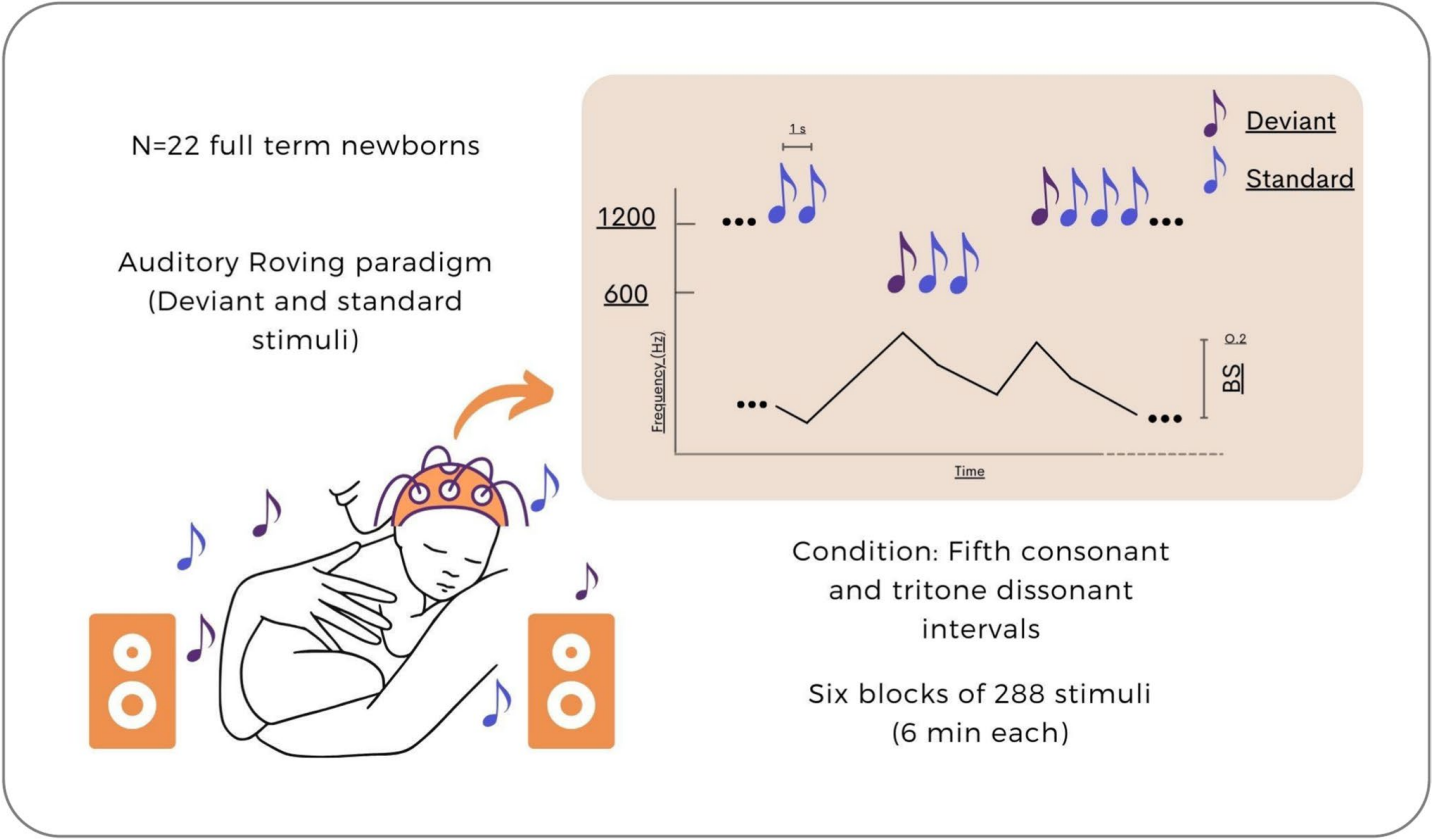
In our roving paradigm, newborns listened to six blocks of 288 sounds. We presented fifth (consonant) and tritone (dissonant) sound intervals separately: Three blocks were composed of sequences of high-pitch and low-pitch consonant intervals, while the other three were composed of sequences of high-pitch and low-pitch dissonant intervals. The top-right graph represents an example of a sequence of standard and deviant sounds. The frequency (Hz) is represented on the *y*-axis. Sound intervals that differed from the preceding one were considered deviant

Newborns were exposed to a standard auditory roving paradigm (overall recording duration: from 30 min to 1 h, depending on the newborn’s compliance), while we recorded their neural activity with a 30-channel electroencephalography (EEG; Gijsen et al., 2021; Grundei et al., 2023). In a roving paradigm, participants are presented with a sequence of standard, repeated tones interrupted by pitch-deviant ones (Ostwald et al., 2012; Sarasso et al., 2022a, 2022b). Unlike classical oddball paradigms, in roving paradigms, each auditory stimulus (either high- or low-pitch intervals in our experiments) has the same probability of occurrence and can represent both a deviant and a standard event, depending on its position in the stimulus sequence (Baldeweg et al., 2004; Grundei et al., 2023; Ostwald et al., 2012; Rosch et al., 2019; see Fig. 1). In this way, the roving paradigm allows us to differentiate deviancy responses from a generic rarity effect that could occur in sequences with an unmatched probability of occurrence between different stimuli.

Newborns listened to three sequences of consonant sounds (fifth intervals) and three sequences of dissonant sounds (tritone intervals), presented in blocks of 6 min each (with trains of 288 stimuli per block). Each sound was a harmonic interval, composed of the simultaneous presentation of two notes; the ratio between these notes determines the degree of consonance (see the Supporting Information for further details). Specifically, to modulate consonance, we presented fifths and tritone intervals sharing the same tonic, as they represent the smallest possible absolute differences in interval size. Previous studies have shown that these intervals can modulate neural responses based on perceived consonance, independently of the absolute frequency of their composing notes (Sarasso et al., 2019, 2021; Schön et al., 2005). Sounds (50-ms duration) of alternating high/low pitch were presented at a frequency of 1 Hz (intertrial interval: 1 s). Note that all sounds included in the sequence had the same probability of occurrence (Fig. 1). For further details about the stimuli and the apparatus, please refer to the Supporting Information.

### EEG preprocessing and statistical analysis

#### EEG preprocessing

An extended version of the EEG analyses is reported in the Supporting Information. Continuous EEG data were segmented into epochs, including 200 ms before stimulation and 800 ms after stimulation (total epoch duration: 1 s), and band-pass filtered (1–30 Hz) using a fast Fourier transform filter in accordance with previous literature exploring ERP responses in newborns (Ronga et al., 2021) and MMN effects and Bayesian surprise in adults (Ostwald et al., 2012; Sarasso et al., 2022a, 2022b). Each epoch was baseline corrected using the interval from 200 to 0 ms. Artifacts due to eye blinks or eye movements were eliminated using a validated method based on an independent component analysis (ICA; Jung et al., 2000). Epochs belonging to the same interval type (i.e., consonant or dissonant) and to the same condition (i.e., standard vs. deviant) were averaged time-locked to the onset of the stimulus, thus yielding four average waveforms (consonant deviant, consonant standard, dissonant deviant, dissonant standard) for each subject. To compute MMRs, the responses to standard-repeated sounds were subtracted from the response to deviant sounds (Näätänen et al., 2007) for each newborn and for each condition (consonant vs. dissonant) separately. Furthermore, we computed trial-by-trial Bayesian surprise values, indexing the informative value conveyed by each sound included in the sequence (for a total of 1,728 Bayesian surprise values). The Bayesian surprise index was computed as the Kullback–Leibler divergence between prior and posterior distributions using a sequential Bayesian learning algorithm of stimulus probabilities (Baldi & Itti, 2010; Grundei et al., 2023; Itti & Baldi, 2009; Ostwald et al., 2012; Sarasso et al., 2022a, 2022b).

#### MMRs to consonant and dissonant sounds

To compare the MMRs elicited by fifth and tritone intervals, we performed a whole-brain, fully data-driven analysis without any a priori assumption by computing a point-by-point *t* test on differential MMRs (fifth vs. tritone). We performed a point-by-point *t* test (Novembre et al., 2018; Sarasso et al., 2021, 2022a, 2022b; Sebastiano et al., 2024), with cluster size-based permutation correction for multiple comparisons based on temporal consecutivity and spatial adjacency (1,000 permutations; alpha level =.05; percentile of mean cluster sum = 95; minimum number of adjacent channels = 3) on differential MMRs (deviant–standard). The test compared single subjects’ MMR amplitudes for fifth and tritone chords at each time point, for each channel separately. This allowed us to identify time clusters containing mismatch detection responses (deviant–standard) that significantly differed between fifth consonant and tritone dissonant intervals.

#### Correlation with Bayesian surprise values

To compute the correlation with Bayesian surprise, we performed a point-by-point Pearson correlation analysis between single-trial preprocessed epochs (i.e., 864 epochs for each condition) and the matrix of Bayesian surprise values corresponding to outputs of the Bayesian surprise algorithm (Sarasso et al., 2021, 2022a, 2022b). The Bayesian indices represent the informational value calculated for each stimulus of the auditory stream (see the Supporting Information for further details), and range between 0.0003 (minimum value) and 0.631 (maximum value). The analysis computed, for each participant, for each EEG channel, and for each condition separately, the Pearson correlation between trial-by-trial fluctuations of the EEG signal and the Bayesian surprise value. Specifically, the Bayesian surprise value of each stimulus was correlated with the corresponding evoked-potential amplitude at each time point. This point-by-point correlation allows us to verify which specific time windows better correlate with Bayesian surprise values and, more specifically, whether these windows coincide with the components mostly contributing to MMRs.

*R* values constituted the input for the subsequent group-level analyses. As a first step, we performed a point-by-point *t* test on each *r*-value time series (consonant and dissonant separately) against the constant zero. This analysis was crucial to verify that the *r*-value time series of consonance and dissonance conditions were significantly different from zero, thus indicating that the neural signal fluctuations actually reflected a form of Bayesian learning. Furthermore, to verify whether the correlation with Bayesian surprise significantly differed across consonance conditions, we performed a two-tailed point-by-point *t* test comparing single subjects’ correlation coefficients, for each condition (consonance vs. dissonance).

In addition to these analyses, we conducted supplementary analyses, using an approach traditionally employed in newborn populations, which further confirmed our results (see section B3 Supplementary Analysis in the Supporting Information).

## Results

### MMRs to consonant and dissonant sounds

#### Response to standard and deviant events and MMRs

**Fig. 2:**
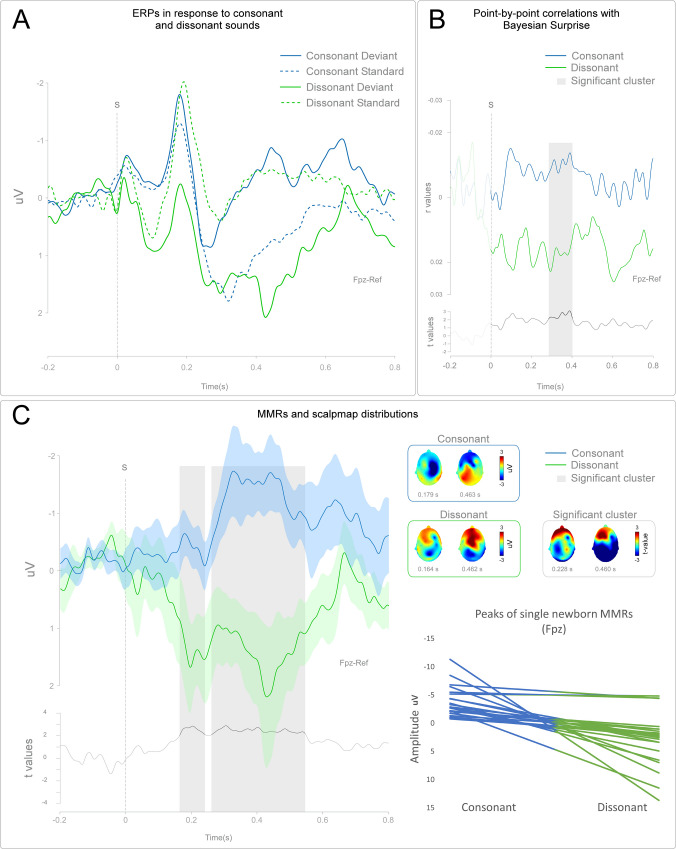
**A** Grand-average ERP registered from Fpz for consonant and dissonant sounds. *x-*axis: time (s); *y-*axis: amplitude (uV). S = stimulus onset. **B** The waveforms represent grand-average *r* values between trial-by-trial neural signal fluctuations registered at Fpz and Bayesian surprise. Shaded areas highlight significant time clusters between conditions (point-by-point statistics). **C** Grand-average MMR waveforms for consonant and dissonant sounds registered on Fpz. Shaded areas highlight significant time clusters between conditions (point-by-point statistics). The scalp maps on the top-right panels show the scalp distribution of MMRs around 200 and 400 ms post-onset and the distribution of the significant difference between MMRs to consonant and dissonant sounds around 200 and 400 ms post-onset. The bottom-right graph shows the peaks (time window: 200–400 ms) of each newborn’s MMRs to consonant and dissonant sounds. (Color figure online)

Newborns’ neural responses elicited by consonant and dissonant intervals recorded from Fpz are reported in Fig. 2A. Exploring the grand-average ERP waveforms before delving into the MMRs may help clarify the origins of the shape, latency, and polarity of the MMR subtraction waveforms, thereby facilitating a better understanding of the results. The grand-average waveforms were consistent with those observed in previous studies on newborns, highlighting distinct responses to deviant versus standard stimuli, within the time window 200–450 ms, over frontocentral electrodes (Friedrich et al., 2009; Garcia-Sierra et al., 2016; Virtala et al., 2013; Werwach et al., 2022; Fig. 2A). Around 200 ms, both deviant and standard dissonant responses show a negative polarity, with standard ERPs exhibiting a greater negative deflection as compared with deviant ERPs. Instead, the deviant consonant curve showed a more pronounced negative polarity as compared with its standard one. In this time window, therefore, MMR polarity is primarily driven by the increased negativity of the consonant deviant curve and the dissonant standard curve. Conversely, between 250 and 550 ms (the time window typically associated with newborn MMRs), the MMR negativity or positivity is mainly driven by the opposite polarity of standard and deviant responses (Fig. 2A). Importantly, MMRs were elicited by both consonant and dissonant intervals, and showed two peaks of opposite polarity at approximately 200 and 400 ms post-onset (Fig. 2C, MMR). The whole-brain point-by-point *t* test revealed that consonant and dissonant MMR waveforms were significantly different in two time windows, between 165 and 245 ms (MMR first peak), and between 260 and 546 ms post-onset (MMR second peak), among frontocentral electrodes. Interestingly, the scalp topographies of consonant and dissonant MMRs also showed clear differences, as revealed by the output of both the point-by-point analysis (Fig. 2C) and the topography analysis of variance (i.e., TANOVA; Koenig & Melie-García, 2010; Wagner et al., 2017), reported in the Supporting Information. Consonant MMRs display a broad frontocentral negativity, with a right-lateralized activity peaking at 200 ms and left-lateralized activity peaking at 400 ms. Dissonant MMRs display a broad and less lateralized positive, frontocentral activity, peaking at 200 and 400 ms.

### Correlation with Bayesian surprise values

The outcome of the point-by-point correlation between trial-by-trial neural signal amplitudes and Bayesian values returned 1-s-long (from 200 ms pre-onset to 800 ms postonset) time series of *r* values for each channel and condition (consonant vs*.* dissonant intervals). Bayesian correlation values reflect the novelty of the presented stimuli, highlighting portions of the curves that significantly encode stimulus information. Since MMR reflects the neural discrepancy between deviant and standard intervals, the two measures (i.e., Bayesian correlation values and MMRs) should be directly correlated (i.e., in amplitude and polarity), as previously shown in adults’ studies (Sarasso et al., 2022a, 2022b). *R* values exhibited specular polarity for consonant and dissonant intervals (see Fig. 2B), reaching their maximum enhancement at around 100 ms, 300 ms, and 400 ms post stimulus onset, which corresponds to the MMR time window.

The time series elicited by consonant intervals were significantly different from zero in the time window between 100 and 400 ms post-onset over the frontoparietal channels, while the* r*-value time series elicited by dissonant intervals were significantly different in the time window around 600 ms over frontocentral channels. Overall, these results show that MMR amplitude fluctuations correlate with Bayesian surprise values, thus confirming that, in newborns too, MMR amplitude indexes perceptual learning.

The point-by-point *t* test performed between conditions revealed that the *r*-value waveform elicited by consonant and dissonant intervals were significantly different in the time interval between 100 and 400 ms (corresponding to the latency of MMRs) over frontocentral electrodes (Fig. 2B).

## Discussion

In the present experiment, we show that, as early as a few hours after birth, newborns exhibit an effective perceptual learning for all the presented sound types, as demonstrated by the combined presence of MMRs and by the trial-by-trial correlation with Bayesian surprise values. Furthermore, newborns displayed two different patterns of neural response, characterized by negative MMR polarity for consonant intervals and positive MMR polarity for dissonant intervals, supporting the hypothesis that two distinct neural mechanisms may be involved in their processing. We speculate that such neural specialization might represent an evolutionarily acquired neural tuning to detect and discriminate salient auditory stimuli, with acoustic features resembling human voice spectra.

### Auditory perceptual learning in newborns

Overall, our findings highlight newborns’ ability to generate and update encodings of auditory regularities according to the informativeness of each new event in the unfolding auditory stream. Previous research exploiting oddball paradigms showed that newborns are able to discriminate stimuli in different sensory modalities within hours of birth (Čeponien et al., 2002; Trainor, 2012; Virtala et al., 2013). Here, we demonstrate that even when the probability of occurrence of each stimulus in the sequence is matched, newborns still display MMRs, which show their ability to detect random, local violations of a pattern.

Furthermore, we showed that the amplitude of trial-by-trial responses to auditory events significantly correlates with Bayesian surprise values, similarly to what is observed in adults (Sarasso et al., 2022a, 2022b). This finding indicates that the amplitude of MMRs in newborns is directly related to the amount of novel information carried by each stimulus in the auditory sequence. To sum up, independently of the specific features by which a stimulus deviates from the auditory stream, newborns seem to display a finely tuned sensitivity to novelty. The combined presence of MMRs, reflecting deviancy encoding, and of a correlation with Bayesian surprise values significantly different from zero (which signals the neural encoding of stimulus informativeness) might be considered as supporting evidence for the presence of a primitive, though effective, form of perceptual learning at birth (Garrido et al., 2009; Perez et al., 2017). In a way, this result is not surprising, given the role that the discrimination of most informative auditory stimuli (such as language) plays in the earliest human developmental stages (Sambeth et al., 2009; Trainor, 2012; Werwach et al., 2022).

### An early mechanism to detect change in salient consonant sounds?

Consonant and dissonant intervals elicited MMRs of opposite polarities, with consonant auditory stimulation triggering negative MMRs, reminiscent of an adult-like mismatch negativity (Näätänen et al., 2007; Sarasso et al., 2021, 2022a, 2022b). This finding suggests the presence of dissociable neural mechanisms dedicated to the processing of consonant versus dissonant stimuli, and this idea is further supported by the scalp distribution of the MMRs (see Fig. 2C). Interpreting our results through the lens of both the *maturation* and *multiple-process* hypotheses (see Introduction) may provide valuable insights into the underlying mechanisms of the present findings (Govaart et al., 2023). Negative MMRs are usually associated with later developmental stages in infants (He et al., 2007; Werwach et al., 2022). However, positive and negative MMRs can coexist in early infancy, with negative MMRs reflecting the mechanism employed to distinguish relevant linguistic stimuli while positive MMRs are often related to more effortful and less specialized acoustic processing (Govaart et al., 2023; Werwach et al., 2022). Interestingly, our original neurocomputational approach provides evidence that, already a few hours after birth, a distinct and more adult-like perceptual learning process can be observed for consonant sounds only. This result is in line with the findings of a previous study using an oddball paradigm to investigate consonance processing (Virtala et al., 2013). The authors showed that newborns’ electrophysiological responses are different for different types of deviant chords (such as a minor or dissonant chord included in a stream of major chords). Importantly, previous behavioral research on gaze orientation (Masataka, 2006) and sound production (Di Stefano et al., 2017) also seems to support the hypothesis of a preferential processing for consonant sounds in newborns. It seems plausible that the adult-like perceptual learning process observed here for consonant stimuli may represent the neural underpinnings of the observed preference for consonant sounds in newborns.

But what might be the adaptive value of a neural tuning for consonance at birth? As we have anticipated, according to the vocal similarity theory, the preference for consonance is explained by the similarity to the human voice spectrum (Bowling et al., 2017). It is possible that, throughout evolution, consonant stimuli resembling human vocalizations acquired a distinct perceptual learning process that is advantageous for language acquisition (Bowling et al., 2017). In other words, consonant patterns in the auditory stream may be marked as salient by newborns’ nervous systems, possibly because they are more likely to originate from conspecifics. This interpretation aligns with, and extends to consonance, the *phonological saliency hypothesis,* which posits that the more phonologically salient a stimulus is, the greater the detected deviance, and the earlier the MMN is observed (Cheng et al., 2015; Hua, 2009). Overall, this finding supports the presence of a clear link between MMRs in early infancy and the later development of the neural and behavioral mechanisms underlying salience detection and language processing (Cheng et al., 2015; Hua, 2009).

The idea of a connection between language development and advanced perceptual learning phenomena seems to be confirmed by other electrophysiological studies on young infants. The effectiveness of auditory perceptual learning in the first months of life has been shown to be a crucial prerequisite for later language development (Garcia-Sierra et al., 2016; Ylinen et al., 2017). Friedrich and colleagues (2009) demonstrated that children displaying age-appropriate expressive language abilities at 2.5 years exhibited an early (at 3–4 months) negative MMR when processing native language deviant stimuli in an oddball design. Conversely, infants developing poorer language skills did not display early negative MMRs (Friedrich et al., 2009). Similarly, the quantity of linguistic input infants receive during their first year of life predicts neural perceptual learning maturity, as indexed by the presence of negative MMRs (Garcia-Sierra et al., 2016).

The difference in the processing of consonant stimuli that we observed, therefore, might be the product of a more refined neural mechanism, possibly developed for early language recognition (Ghio et al., 2021). Dissonant stimuli, on the other hand, could be processed by a more basic pathway, devoted to general, less salient, acoustic input (Govaart et al., 2023; Ylinen et al., 2017). An extensive literature confirms that humans, even during intrauterine life, discriminate voice and speech sounds and display an early left-hemispheric specialization for linguistic processing (Draganova et al., 2007; Ghio et al., 2021). Interestingly, even in the present study, the scalp distribution is significantly different (see SI), with MMRs elicited by consonant intervals displaying left lateralization over frontocentral electrodes, whereas dissonant intervals eliciting a more widespread topographical distribution in the parieto-central electrodes (Fig. 2C). If further confirmed by future studies exploring the source localization of MMRs, this finding might therefore be considered as supporting evidence for the involvement of a primitive language-related pathway in consonant stimulus processing.

### Nature or nurture?

Our study could contribute to the ongoing discussion about the respective roles of biology (nature) and cultural exposure (nurture) in determining consonance and dissonance perception (Bowling et al., 2017), an issue strongly debated in musicology, auditory psychology, and neuroscience. Our results, indicating an early tuning for consonance sensory processing, seem to lend support to the “nature” explanation. Nevertheless, it is important to note that such early neural tuning toward consonance could be shaped during the gestational period, suggesting the need for further investigation during intrauterine life. In any case, it seems evolutionarily adaptive to display a preferential neural pathway to process stimuli belonging to one’s own species’ vocalizations or at least with the same acoustic spectra. By directly comparing studies using the same methodologies to explore consonance and dissonance perceptual encoding in adults and infants (Sarasso et al., 2021, 2022a, 2022b), we propose that at birth, newborns are provided with a preferential and more adult-like neural mechanism to process consonant input. The previously described dedicated deviancy-detection (early nMMR) for phonologically salient stimuli (Cheng et al., 2015), seems to be extended here to acoustic stimuli that resemble conspecific communication. This would broaden the range of potentially relevant auditory inputs at a developmental stage in which the language system is not yet fully mature. At a later developmental stage, experience could shape and refine this process and perhaps tune infants, for example, toward native language sounds or phonologically relevant information. Throughout development, adults might eventually devote this preferential pathway (signaled by magnified mismatch negativity responses) to stimuli that have come to be considered more salient, either by experience or by nature. A previous study using the present experimental paradigm in adults (i.e., comparing MMN responses elicited by a roving paradigm composed of a consonant and dissonant interval) showed that, while most participants exhibit subjective preference and neural magnification for consonance, one out of five participants displayed an identical neural tuning (and aesthetic preference) to dissonant sounds, with a magnification of the mismatch negativity for these stimuli (Sarasso et al., 2021, 2022a, 2022b). We speculated that such a neural tuning for dissonant stimuli, rather than innate, was gradually developed in response to specific life experiences (Brattico & Jacobsen, 2009; Brattico et al., 2013; Crespo-Bojorque et al., 2018; Jacoby et al., 2019; Lahdelma & Eerola, 2020; Mencke et al., 2019; Weiss et al., 2020). It is worth noting that neural tuning in adults is systematically associated with an aesthetic reward (Sarasso et al., 2020, 2021), since greater MMRs are consistently correlated with subjective aesthetic preferences (Sarasso et al., 2021, 2022a, 2022b). Future studies might therefore explore the emergence and development of an explicit aesthetic preference throughout infancy.

### Conclusions and future directions

In conclusion, our results suggest that newborns exhibit electrophysiological correlates of auditory perceptual learning processes within hours of birth and that these processes differ for consonant and dissonant stimuli. A specific neural process seems to be dedicated to the processing of novel, consonant stimuli, providing newborns with an early mechanism to discriminate salient information in the noisy auditory environment, likely facilitating language acquisition.

Further evidence will be needed to confirm this hypothesis, to empirically test the interpretation of our findings (particularly in relation to the vocal similarity theory), and to further clarify the developmental trajectory of perceptual learning processes throughout infancy. Moreover, future studies should be directed to further investigate the neural encoding of sensory regularities in newborns and other possible factors that might modulate it. As an example, it may be important to investigate the role of different physical and contextual features, such as the presence versus absence of social interaction, in modulating perceptual learning. Finally, it would be interesting to investigate how such neural mechanisms emerge within ontogeny from the intrauterine life to early infancy.

## Supplementary Information

Below is the link to the electronic supplementary material.

Supplementary file1 (DOCX 39 kb)

## Data Availability

Raw data used in the study and the tables containing the mean amplitude used in the analysis are fully available at this link (Mendeley, https://data.mendeley.com/preview/8vcx3ygxtw?a=fd2fe388-7524-404a-b37b-99b65cf0438c). All the data collected for the present research will be made publicly available upon acceptance for publication.

## Code availability

All codes, procedures, and software used in the study are available at Mendeley link (https://data.mendeley.com/preview/8vcx3ygxtw?a=fd2fe388-7524-404a-b37b-99b65cf0438c).
