# Supplementary material for: This sounds important: Electrophysiological responses reveal a dedicated learning mechanism to process salient consonant sounds in human newborns
Source: Psychon Bull Rev. 2026 Jun 15;33(5):177. doi: 10.3758/s13423-026-02911-w (PMC13269445; doi:10.3758/s13423-026-02911-w)
Supplement: Supplementary file 1 — Supplementary file1 (DOCX 39 kb) [file 13423_2026_2911_MOESM1_ESM.docx]

**Supporting Information**

.

**Table of Contents**

**A Method**

**A1 Participants**

**A2 Stimuli and apparatus**

**A3 Electroencephalogram recording and processing**

**A4 Roving paradigm and Procedure**

**A5 Bayesian Perceptual Surprise Computation**

**B Data Analysis**

**B1**  **Statistical analyses on MMRs**

**B2 Correlation with Bayesian surprise values**

**B3 Supplementary Analysis**

**C Results**

**C1 MMRs Results**

**C2 Trial-by-Trial Correlation with Bayesian Surprise**

**C3 Supplementary results**

**A Method**

**A1 Participants**

22 full term healthy newborns (females: 10; gestational age > 37 weeks) participated in the Experiment conducted at the obstetric Hospital, where they were born. Inclusion criteria were identical to those of the the American College of Obstetricians and Gynecologists (ACOG), i.e., birth weight > 2500 g, weeks, and Apgar index score = 9 at 5 minutes of life. No evident abnormalities were recorded at birth in the participants. The 22 newborns included in the final sample had a mean age of 40.4 hours at the time of testing (*SD* = 15.8), a mean birth weight of 3.267 g (*SD* = 361), and a mean gestational age at birth of 38,9 weeks and 3 days.

All newborns’ parents were fully informed about the experimental procedures prior to the experiment and they gave their written informed consent to allow the study. The study conformed to the standards required by the Declaration of Helsinki and was approved by the local ethics committee. The original sample size (*N* = 22) was a priori determined to match the average number of participants involved in previous ERP studies on newborns (Ronga et al., 2021). Newborns included in the final dataset (N = 22) were awake, relaxed in their mothers’ arms, who were comfortably sitting on an armchair. The experiment could last between 30 minutes and an hour for the situational needs of newborns; indeed, we aimed at testing them when they were awake and relaxed, to wait for the best moment to collect the data.

**A2 Stimuli and Apparatus**

Intervals were created with Csound (<https://csound.com/>), which allows to select the frequency of the two notes composing the interval, which were played simultaneously for 50 ms via a loudspeaker. Loudness of sounds was kept equal across subjects and set at a comfortable level (64 dB). Each presented sound was a harmonic interval, composed of the simultaneous presentation of two notes; the ratio between these notes determines the degree of consonance. Specifically, the ratio between the frequency of the two composing notes defined two interval types (i.e., Fifth consonant and Tritone dissonant): the smaller the integer numbers that define the ratio, the more consonant the interval (Fishman et al., 2001; Plomp & Levelt, 1965). Perfect fifth intervals, usually perceived as more consonant by adults, have a ratio of 3:2, while tritones, frequently categorised as more dissonant, are defined by a ratio of 45:32. For each interval type (fifths or tritones), we employed two different pitches (i.e., high vs low). Low-pitch and high-pitch fifth intervals were composed of notes with a frequency of 150 and 100 Hz and 600 and 400 Hz, respectively. Low-pitch and high-pitch tritone intervals were composed of notes with a frequency of 150 and 106 Hz and 600 and 426 Hz, respectively. During the experiment, newborns were relaxed on their mothers’ lap and the loudspeaker was placed 20 cm from them.

**A3 Electroencephalogram recording and processing**

Newborns’ electrophysiological neural responses to the auditory roving paradigm were recorded using 32 Ag-AgCl electrodes placed on the scalp according to the International 10-20 system and referenced to the nose. Electrode impedances were kept below 5 kΩ. The electro-oculogram (EOG) was recorded from two surface electrodes, one placed over the right lower eyelid and the other placed lateral to the outer canthus of the right eye. Signals were digitized at a sampling rate of 2048 Hz with a Handy EEG amplifier (SystemPlus Evolution, Micromed, Treviso, Italy). EEG data were offline pre-processed and analysed using Matlab (Mathworks, Natick, MA). Continuous EEG data were segmented into epochs including a 200 ms before stimulation and 800 ms after stimulation (total epoch duration: 1 s), and band-pass filtered (1-30 Hz) using a fast Fourier transform filter (in accordance with previous literature exploring ERP responses in newborns and MMN and Bayesian surprise in adults (Ostwald et al., 2012; Ronga et al., 2021; Sarasso, Barbieri, et al., 2022). Each epoch was baseline corrected using the interval from -200 to 0 ms from the stimulus trigger. Artifacts due to eye blinks or eye movements were eliminated using a validated method based on an Independent Component Analysis (ICA; Jung et al., 2000). Epochs belonging to the same interval type (i.e., fifth or tritone) and to the same condition (i.e., standard vs. deviant) were averaged time-locked to the onset of the stimulus trigger, thus yielding four average waveforms (Fifth Deviant, Fifth Standard, Tritone Deviant, Tritone Standard) for each subject.

**A4 Roving paradigm and Procedure**

Newborns passively listened to six runs of a standard roving paradigm with trains of 288 stimuli per run (overall recording duration: from 30 minutes to 1 hour, depending on the newborn’s compliance), while we registered their neural activity with electroencephalography (EEG). Three runs of only the fifth consonant (high-pitch and low-pitch) and three runs of only tritone dissonant (high-pitch and low-pitch) intervals were presented. The order of presentation of the fifth and tritone runs was randomised across participants to exclude any specific sequence effects. In roving paradigms, in contrast to traditional oddball (Näätänen et al., 2007), different stimuli (high-pitch and low-pitch in our experiment) represent both Deviant and Standard stimuli (Baldeweg et al., 2004; Ostwald et al., 2012; Rosch et al., 2019; Sarasso, Neppi-Modona, et al., 2022), and each stimulus has exactly the same probability to occur, thus allowing us to dissect the genuine effects of Bayesian perceptual learning from rarity-driven modulations (Sarasso, Neppi-Modona, et al., 2022). During the experiment, newborns were presented with consecutive trains of high and low pitch intervals with a constant interstimulus interval of 1 s (Sarasso, Neppi-Modona, et al., 2022); any time a particular stimulus (low pitch or high pitch) repeats itself constitutes a sequence of Standard stimuli, while when the stream of standard sound is interrupted by a different stimulus (low or high pitch), the first stimulus of the new train constitutes a Deviant event, since it differs (by its pitch) from the preceding train of Standard stimuli (McCleery et al., 2019; Sarasso, Neppi-Modona, et al., 2022). Similarly, the repetition of the Deviant stimulus constitutes a new sequence of standard, repeated intervals. A pseudorandom order was used to constitute the length of the train of high and low-pitch intervals, so that both the number of presentations and the average value of the Bayesian surprise associated with each stimulus were equal across interval type (i.e., fifths or tritones) and pitch type (i.e., high or low). Moreover, the ratio between Standard (80%) and Deviant (20%) trials was kept equal across runs.

**A5 Bayesian Perceptual Surprise Computation**

We computed the Bayesian surprise for each trial under a Beta-Binomial model of Bayesian learning of the stimulus probabilities (Baldi & Itti, 2010; Itti & Baldi, 2009; Ostwald et al., 2012; Sarasso, Neppi-Modona, et al., 2022). The model assumes that the brain implements a sequential learning procedure starting from an uninformative prior, updates this prior according to the subsequent observations, and computes Bayesian surprise as the Kullback-Leibler divergence between prior and posterior. Following Ostwald and colleagues (Ostwald et al., 2012), we use a variant of this model that assumes an exponential forgetting of stimuli that are observed in the distant past (Kullback, 1959; Sarasso, Neppi-Modona, et al., 2022). Formally, we assume that the probability of observing a low (x = 0) or high (x = 1) intensity stimulus at a given trial is described by a Bernoulli distribution with parameter µ ∈ [0, 1]:

Pr(x|µ) = µ^x^ (1 − µ)^1-x^, x ∈ {0, 1} .

The true value of µ is unknown by the subject, and the initial uncertainty is modelled by means of an informative prior of type Beta, whose density function is uniform on the unit interval:

f (µ) = 1, ∀µ ∈ [0, 1] .

On each trial, the prior is sequentially updated according to the observed data likelihood to form a posterior distribution over µ. After N trials, let x^N^ denote the sequence of observed stimuli

$$x^{N}= \left[ x1 \vdots xN \right]$$

where x_i_ ∈ {0, 1} for each *i* = 1, ..., N. Under standard Bayesian learning, the posterior over µ is computed as follows. The probability of observing a x^N^ when the true parameter is µ is:

Pr ( x^N^ |µ) = $\mu^{n_{N}}{(1 - \mu)}^{m_{N}}$

Bayes rule implies that the posterior over µ is a Beta distribution with density

f (µ|x^N^) = $\frac{PrPr (x^{N}| \mu) f( \mu)}{\int_{0}^{1} Pr (x^{N}|\nu) f \left( \nu\right) d\nu}$ = $\frac{\mu^{n_{N}} {(1- \mu)}^{m_{N}}}{B (1 + nN , 1+mN)}$

where n_N_ = |{i : x_i_ = 1}| is the number of high-intensity stimuli, m_N_ = N – n_N_ is the number of low-intensity stimuli, and B is the Beta function. In order to account for a forgetting dynamic, instead of using the accumulative stimulus counts n_N_ and m_N_ in the previous formula, the model employed here weights past observations according to an exponential function. Define the weighted stimulus counts $n_{N}^{\tau}$ and $m_{N}^{\tau}$ by

$$n_{N}^{\tau}:= \sum_{i=1}^{N} exp\left( -\frac{1}{\tau} \left( N-i \right) \right) x_{i}.$$

And

$$m_{N}^{\tau}:= \sum_{i=1}^{N} exp\left( -\tau\left( N-i \right) \right) \left( 1- x_{i} \right).$$

τ ≥ 0 is a parameter governing the forgetting dynamics: for τ = 0 we have $n_{N}^{\tau}$ , $m_{N}^{\tau}$ = n_N_ , m_N,_ whereas increasing the value of τ implies that past observations are weighted less and less. This results in a posterior density given by

$$f^{\tau}\left( \mu| x^{N} \right)= \frac{x^{n_{N}^{\tau}} \left( 1- \mu\right)^{m_{N}^{\tau}}}{B\left( 1+ n_{N}^{\tau}, 1+ m_{N}^{\tau} \right)}.$$

Finally, the model quantifies the degree of learning as the Bayesian surprise, or Kullback–Leibler divergence, between the prior and posterior distribution over µ after a given trial. Let x denote the full sample of observed stimuli. The Bayesian surprise after the N-th trial is given by:

$${Surprise}_{N}^{\tau}\left( x \right)\equiv KL\left( f^{\tau}\left( \cdot|x^{N} \right)| f^{\tau}\left( \mu|x^{N-1} \right) \right)$$

$$\equiv\int_{0}^{1} f^{\tau}\left( \mu|x^{n-1} \right) loglog \frac{f^{\tau}\left( \cdot|x^{N-1} \right)}{f^{\tau}\left( {\mu| x}^{N} \right)} d\mu.$$

Due to the use of conjugate priors, the Kullback–Leibler divergence can be evaluated analytically, which significantly simplifies the computation (Kullback, 1959).

**B Data Analyses**

**B1 Statistical analyses on MMRs**

To compare newborns' neural response in sensory processing of fifth consonant and tritone dissonant intervals, we computed the mismatch response analysis (i.e. MMR). More specifically, we computed the MMR by subtracting the average ERP response elicited by Standard intervals from the average ERP response elicited by Deviant Intervals for each newborn and each condition (i.e., Fifth and Tritone chords; Näätänen et al., 2007). Crucially, we performed the analysis considering only the last interval of the standard sequence (i.e., the one before deviant trials) so as to match the number of standard and deviant trials (*N* = 52 per run; Ostwald et al., 2012; Sarasso, Neppi-Modona, et al., 2022).

*Group level statistical analyses.* To compare the MMR elicited by Fifth and Tritone intervals, we performed a whole-brain fully data-driven analysis without any aprioristic assumption by computing a point-by-point t-test on differential MMR (Fifth vs Tritone). We performed a point-by-point t-test (Novembre et al., 2018), with cluster size-based permutation correction for multiple comparisons based on temporal consecutivity and spatial adjacency (1,000 permutations; alpha level = .05; percentile of mean cluster sum = 95; minimum number of adjacent channels = 3) on differential MMR (Deviant–Standard). The test compared single subjects’ MMR amplitudes for Fifth and Tritone chords at each time point, for each channel separately. This allowed us to identify time clusters containing mismatch detection responses (Deviant–Standard) that significantly differed between Fifth Consonant and Tritone Dissonant intervals.

**B2 Correlation with Bayesian surprise values**

The preprocessing steps in the present analysis were identical to the MMR analysis, with the only substantial difference being that all trials must be maintained to compute the correlation between amplitudes and the Bayesian Surprise index. Therefore, we excluded participants with fewer than 80% trials that survived the artifact correction. Four participants were excluded from the correlation analysis after visual inspection. However, the remaining sample (*N* = 18) displays an ERP response to the roving paradigm identical to that of the original sample (*N* = 22), as demonstrated by a significant difference in the polarity of the MMRs elicited by consonant and dissonant intervals (see the Results section below for further details). First, a point-by-point trial-by-trial Pearson correlation analysis between preprocessed epochs (i.e., 864 for each condition) and the matrix of Bayesian surprise values (i.e., minimum value = 0.0003, maximum value = 0.631) corresponding to single trials (i.e., 864 for each condition; (Novembre et al., 2018; Sarasso et al., 2019, 2021; Sarasso, Neppi-Modona, et al., 2022) was performed. The analysis computed, for each participant, for each EEG channel and for each condition separately (i.e., Fifth consonant and Tritone dissonant intervals), the Pearson correlation between trial-by-trial fluctuations of the EEG traces and the Bayesian surprise value. The outcome of the correlation analysis was two 1s-long (from 200 ms preonset to 800 ms postonset) time series of r-values for each channel and for each subject.

*Group level statistical analyses.* To verify that EEG amplitudes reflected the Bayesian updating as measured by the Bayesian Surprise index, we performed a point-by-point t-test on each r-value time series (Fifth and Tritone separately) against the constant zero. The two 1 s-long (from 200 ms preonset to 800 ms postonset) time series of r-values for each channel, for each subject and each condition (Fifth and Tritone) separately, constituted the input for the subsequent group-level two-tailed point-by-point t-test with permutation-based correction for multiple comparisons (1,000 permutations; alpha level = .05; percentile of mean cluster sum = 95; minimum number of adjacent channels = 3) against the constant zero. The test compared single subjects’ (*N* = 18) correlation coefficients, for each condition separately (Fifth and Tritone) against the constant zero at each time point.

Furthermore, as in the case of the MMRs analysis, to compare the differences between the r-values time series elicited by consonant and dissonant intervals (Fifth vs Tritone), we perform a point-by-point t-test on the differential r-values time series (Fifth vs Tritone). The time series of r-values for each channel and for each subject constituted the input for the subsequent group-level two-tailed point-by-point t-test with permutation-based correction for multiple comparisons (1,000 permutations; alpha level = .05; percentile of mean cluster sum = 95; minimum number of adjacent channels = 3). The test compared single subjects’ (*N* = 18) correlation coefficients for each condition (Fifth vs. Tritone) at each time point. This allowed us to identify time clusters containing r-values that significantly differed between Fifth consonant and Tritone dissonant intervals.

**B3 Supplementary analysis**

Given the wide variability of methodologies and results found in the literature, we confirmed our analyses with less conservative approaches that are more commonly used in studies on newborns.

For the supplementary analysis of both MMR and Bayesian Surprise, we selected a time window that is broader than those typically used in comparable studies conducted with adults. The 200–450 ms time window was chosen based on previous literature on the oddball paradigm in newborns and young infants, which reports a peak response within this interval (Chen et al., 2022; Cheng et al., 2015; Cheour et al., 2002; Dehaene-Lambertz & Baillet, 1998; Dehaene-Lambertz & Dehaene, 1994; Friedrich et al., 2009; Kostilainen et al., 2018; Virtala et al., 2013; Werwach et al., 2022), as well as considering the exploratory nature of employing a roving paradigm in this population.

*MMRs’ mean amplitude comparison*

We performed a “single-point” traditional analysis widely used in newborn studies (Ronga et al., 2021), comparing the mean amplitude (Fifth vs Tritone) recorded in a time window and scalp distribution of interest for the research proposal. More specifically, single newborns’ MMR registered on a single channel were entered in a group-level analysis to test for possible differences in MMR elicited by fifth consonant and tritone dissonant chords. Based on previous literature (Chen et al., 2022; Cheng et al., 2015; Cheour et al., 2002; Friederici et al., 2002; Friedrich et al., 2009; Garcia-Sierra et al., 2016; Kostilainen et al., 2018; Ronga et al., 2021; Virtala et al., 2013; Werwach et al., 2022) on newborns MMR, we calculated the mean amplitude within the 200 - 450 ms range over frontocentral electrodes (FPz, Fz, FP1, FP2, Cz, CP1) for each condition separately (Fifth and Tritone). Specifically, we chose this specific latency window because MMRs in newborns and young infants are typically reported in the literature as peaking between 200 and 400 ms (Chen et al., 2022; Cheng et al., 2015; Cheour et al., 2002; Dehaene-Lambertz & Baillet, 1998; Dehaene-Lambertz & Dehaene, 1994; Friedrich et al., 2009; Garcia-Sierra et al., 2016; Kostilainen et al., 2018; Virtala et al., 2013; Werwach et al., 2022). We then performed a two-tailed t-test between the mean amplitude extracted on the frontocentral electrodes corresponding to the Fifth and Tritone conditions (Ronga et al., 2021).

For the present research, we selected a time window between 200 and 450 ms and a fronto-central region of interest, since MMRs in newborns are usually recorded at these latencies and scalp distribution.

*Group-level ANOVA* *on MMRs.*

We computed a 2 x 2 ANOVA on the average ERP of each participant to further clarify the role of *consonance* and *deviancy* factors and their interaction. Specifically, we computed the statistic on the event-related waveforms of each participant. We set consonance (consonant and dissonant level) and deviancy (deviant and standard level) as a two-level within-factor. The statistical analysis corrects for multiple comparisons using a permutation-based correction (1,000 permutations; alpha level = .05; percentile of mean cluster sum = 95; minimum number of adjacent channels = 3).

*Bayesian surprise’s mean amplitude comparison*

We compared the mean amplitude of the r-values time series in the 200 – 450 ms range (MMR time interval of interest), over the frontocentral electrodes, for each condition separately, against the constant zero. Previous studies suggest that mismatch negativity and Bayesian surprise correlation’ peaks share a common time window and scalp distribution, therefore we extracted the mean r-values in the same cluster of electrodes and in the same time window of the MMR (Fpz, Fz, Fp1, Fp2, Cz, Cp1). To compare the r-values time series using parametric tests (one-sample t-test), we applied Fisher’s r-to-z transformation (Fisher, 1915). We then performed a one-sample t-test to compare mean z-values against the constant zero for each condition (Fifth and Tritone) separately.

We finally compare the mean of the z-values time series previously calculated (200 – 450 ms range and over frontocentral electrodes) by computing a two-tailed t-test (Fifth vs. Tritone). The test compared single subjects’ (*N* = 18) mean z-values time series, for each condition (Fifth vs. Tritone).

*Topographic analysis of variance (TANOVA)*

To compare the scalp topography of newborns’ MMRs, we performed a Topographic analysis of variance (i.e., TANOVA), a well-established, reference-free method for assessing differences in EEG spatial configurations (Koenig et al., 2011; Koenig & Melie-García, 2010; Wagner et al., 2017). Prior to statistical analysis, individual EEG data were normalized across electrodes by dividing each scalp map by its corresponding Global Field Power (GFP) at each time point. This normalization step removes amplitude-related effects and ensures that only differences in spatial configuration (topography) are assessed. At each time point, we computed the squared Euclidean distance between the average normalized maps of the two experimental conditions, providing a time-resolved measure of topographic dissimilarity. To statistically evaluate these differences, we applied a permutation test: the condition labels were randomly shuffled across participants 1000 times, and the TANOVA statistic was recalculated for each permutation. This generated a null distribution against which the observed TANOVA values were compared. The resulting p-value time series indicated, for each time point, whether the scalp topographies differed significantly between conditions.

**C Results**

**C1 MMRs to consonant and dissonant sounds.** The whole brain fully data-driven point-by-point t-test performed on mismatch detection responses registered on Fpz (Fifth vs. Tritone) revealed two significant time clusters: the first one centred on the average MMR waveform peak (165 – 245 ms; Figure 2 panel C); the second one occurring later at 260 –546 ms post onset. As expected, MMR waveforms elicited by Fifth and Tritone intervals were significantly different (*t* _(21)_ = 2.89; *p* = .008 on Fpz). Results were comparable among fronto-central electrodes; the significant cluster corresponding to the MMR extended over Fp1, Fpz, Fp2, F7, F3, and Fz.

**C2 Correlation with Bayesian Surprise values**

The correlation analysis between single-trial amplitudes and Bayesian Surprise indicated that r-values were enhanced at 100 ms (corresponding to the peak of the auditory-evoked potential; Figure 2 B in the main text) for both Fifth and Tritone intervals. Subsequent fluctuations in r-values were enhanced at 300 ms and 400 ms postonset corresponding to the P3 and the N400 components (see Figure 2 B in the main text). These components and time intervals contribute significantly to the composition of the MMR (obtained by subtracting standard from deviant event-related responses), as already demonstrated in adults’ literature (Sarasso, Neppi-Modona, et al., 2022). Thus, these results confirmed our prediction, indicating that MMR best indexes Bayesian perceptual learning in our study. The whole brain point-by-point t-test against the constant zero revealed that both the Fifth consonant and Tritone dissonant time series of r-values resisted the 1000 permutations and were significantly different from zero. More specifically, the time series of r-values elicited by fifth consonant interval were significant in the time window between 100 and 400 ms postonset over frontocentral channel (F4 and P3; *t_(17)_* = -3.25 and *p* = .004 on F4, where the difference in maximal) while the r-values time series elicited by tritone intervals were significantly different in the time window around 600 ms over frontocentral channel (Fpz, F7, F3, Fc1 and Cz; *t_(17)_* = 2.98 and *p* = .008 on Fpz where the difference is maximal).

The point-by-point t-test performed between the r-values time series of the fifth and tritone conditions revealed that the two r-values waveforms (Fifth vs. Tritone) were significantly different in the time interval between 100 and 400 ms (Corresponding to the latency of MMR) over frontocentral electrodes (Fpz, Fp2, F4, Fc1, Fc2). More specifically, the first significant cluster is found in the time interval between 60 and 130 ms over Fp2 (*t_(17)_* = 3.02; *p* = .006) while the second larger cluster is found in the time interval between 280 and 400 ms over Fpz, Fp2, F4, Fc1, Fc2 (*t_(17)_* = 3.1 and *p* = .006 on Fpz where the difference is maximal).

**C3 Supplementary Results**

**MMRs’ mean amplitude results**

The two-tailed paired sample t-test performed on mean amplitudes over the frontocentral electrodes revealed a significant difference between the consonant and dissonant mismatch response, further confirming the point-by-point results (*t_(21)_* = -2.22; *M* = -1.49; *SD* = .67; *p* = .028).

The 18 newborns tested for correlation analysis showed a significant difference in MMRs elicited by consonant and dissonant intervals, identical to the original sample (*N* = 22). More specifically, the point-by-point t-test comparing MMRs elicited by consonant and dissonant intervals revealed a significant cluster in the time interval between 260 and 480 ms among the frontocentral electrodes: Fp1, F7, F3, Fz, Pz (*t_(17)_*= 4.61; *p* = < .001; on Fz where the difference is maximal).

**ANOVA results on MMRs**

*Group level 2 x 2 ANOVA*. The point-by-point ANOVA revealed a significant effect only of the interaction between consonance and deviancy, reflecting the results on mismatch responses described above. More specifically, the statistical comparison revealed a cluster of significance (i.e., effect is maximal on Fpz: *F_(21)_* = 3.51; *p* = .009) of interaction (consonance x deviancy) in the time interval between 160 and 550 ms in frontocentral electrodes (Fp1, Fpz, Fp2, F7, F3, Fz).

**Bayesian surprise’s mean amplitude results**

The One sample t-test performed on the mean amplitude extracted over the frontocentral electrodes (FPz, Fz, FP1, FP2, Cz, CP1) comparing Fifth consonant (*t_(17)_* = -2.00; *M* = -.06; *SD* = .31; *p* = .048) and Tritone dissonant (*t_(17)_* = 3.42; *M* = .12; *SD* = .35; *p* < .001) conditions against the constant zero for each subject reveal a significant difference between the consonant and dissonant z-values time series, further confirming the point by point results.

The two-tailed paired sample t-test performed on the mean amplitude extracted on the frontocentral electrodes comparing Fifth consonant and Tritone dissonant conditions (Fifth vs Tritone) reveals a significant difference between the consonant and dissonant z values time series (*t_(17)_* = -4.09; *M* = -.176; SD = .043; *p* < .001), further confirming the point-by-point results.

**TANOVA results**

The output of the TANOVA analysis was a time-resolved vector of 2048 p-values, corrected for multiple comparisons using a permutation approach. These p-values reveal the time-points at which the scalp topography of newborns significantly differed between conditions. Notably, the analysis highlighted a significant cluster (*p* < .05) of topographic differences in the time window between 280 and 620 ms, further supporting our findings.
